# Joining of Hypereutectic Al-50Si Alloys Using Lead-Free Brazing Filler Glass in Air

**DOI:** 10.3390/ma13245658

**Published:** 2020-12-11

**Authors:** Zhenjiang Wang, Zeng Gao, Xianli Ba, Junlong Chu, Peng He, Jitai Niu

**Affiliations:** 1 School of Materials Science and Engineering, Henan Polytechnic University, Jiaozuo 454003, China; 111706010003@home.hpu.edu.cn (Z.W.); 211806020036@home.hpu.edu.cn (X.B.); 211806020009@home.hpu.edu.cn (J.C.); jtn@hit.edu.cn (J.N.); 2 State Key Laboratory of Advanced Welding and Joining, Harbin Institute of Technology, Harbin 150001, China; hepeng@hit.edu.cn; 3 Henan Jingtai High-Novel Materials Ltd. of Science and Technology, Jiaozuo 454003, China

**Keywords:** hypereutectic Al-50Si alloys, brazing filler glass, diffusion transition layer, crystal phase

## Abstract

Hypereutectic Al-Si alloys are attractive materials in the fields of electronic packaging and aerospace. A Bi_2_O_3_-ZnO-B_2_O_3_ system lead-free brazing filler glass was employed to braze hypereutectic Al-50Si alloys in air. The hypereutectic Al-50Si alloys were pre-oxidized and the low-temperature glass powder was flake-shaped in the brazing process. The effects of brazing temperature and time on joints microstructure evolution, resulting mechanical strength, and air tightness were systematically investigated. The results indicated that the maximum shear strength of the joint was 34.49 MPa and leakage rate was 1.0 × 10^−10^ Pa m^3^/s at a temperature of 495 °C for 30 min. Crystalline phases, including Bi_24_B_2_O_39_ and Bi_2_O_3_, were generated in the glass joint. The formation of a diffusion transition layer with a thickness of 3 μm, including elements of Al, Si, Zn, Bi, Na, and B, was the key to form an effective joint. The elements of Al, Si, and Bi had a short diffusion distance while the elements of Zn, Na, and B diffused in a long way under brazing condition.

## 1. Introduction

With the rapid development of electronic information technology, the integrated circuits of electronic devices require high power, high integration, and miniaturization [1,2]. However, the strict requirements of heat dissipation and light weight have been becoming bottleneck problems restricting the development of high-power electronic devices. As a consequence, the corresponding electronic packaging materials will inevitably face upgrading. Traditional electronic packaging materials include the first-generation material Kovar and the second-generation material W/Cu and Mo/Cu alloy [2]. Kovar alloy, as the most commonly used electronic packaging material, has been used for a long time and played a huge role in electronic packaging in the past for its similar linear expansion coefficient to borosilicate hard glass. However, it has the disadvantages of poor thermal conductivity and heavy weight. Similarly, W/Cu or Mo/Cu alloys showed good thermal conductivity while they were heavy weight and high cost. It can be seen that traditional electronic packaging materials cannot simultaneously meet the requirements of heat dissipation and lightweight. They are increasingly difficult to keep up with the pace of modern electronic packaging. Silicon carbide particle-reinforced aluminum metal matrix composites (SiC_p_/Al MMCs) and hypereutectic Al-Si alloys have become popular candidates for a new generation of electronic packaging materials for their outstanding advantages such as high thermal conductivity, low density and low adjustable thermal expansion coefficient. They have potential applications in aerospace, automobiles, electronics, optical instruments, and sporting equipment, etc. [3,4,5,6]. In particular, for the T/R module of phased array radar, the weight of the phased array radar can be greatly reduced when hypereutectic Al-Si alloys are used to replace the commonly used Kovar alloy [7,8], which has important economic value. High-quality joining of the two materials is a key factor for the practical application of these materials.

In recent years, SiC_p_/Al MMCs have been widely studied, and its preparation process has been relatively mature. However, the joining problem of SiC_p_/Al MMCs has always been a research focus. As the chemical properties of silicon carbide particles are quite different with the base aluminum alloy, the joining of SiC_p_/Al MMCs has been a difficult point. Joining methods for SiC_p_/Al MMCs include flux-free diffusion joining [4], transient liquid phase bonding (TLP) [9], vacuum brazing [10], ultrasonic brazing [11], friction stir welding [3], mechanical vibration agitation brazing [12,13] and semi-solid welding [14,15], etc. Among the above-mentioned joining methods, brazing has good joining effect and economic efficiency. It can realize the joining of SiC_p_/Al MMCs to a certain extent, however, the brittleness and high hardness of SiC_p_/Al MMCs make it extremely difficult to machine. Ordinary tools are difficult to process and shape for it, which limits their practical application in engineering. Therefore, as a substitute, hypereutectic Al-Si alloys which have better weldability and machinability than SiC_p_/Al MMCs has attracted people’s attention. Hypereutectic Al-Si alloys are also called high-silicon aluminum alloys. At present, the research on hypereutectic Al-Si alloys mainly focuses on preparation methods. The British Osprey company took the lead in successfully preparing silicon aluminum alloy materials by spray deposition and hot isostatic pressing. By controlling the silicon content, a series of silicon-aluminum alloy materials called CE alloys (Control Expansion alloys) with a thermal expansion coefficient of (7~17) × l0^−6^/K were formed [16,17]. The current preparation methods mainly include rapid solidification [18], twin roll casting technique [19], electromagnetic directional solidification(EMDS) [20], powder metallurgy [21], spray deposition [22,23,24,25], semi-solid thixoforming [26] and selective laser melting (SLM) [27], etc. However, less research has been carried out on the joining of hypereutectic Al-Si alloys. At present, the research reports on the joining methods of hypereutectic Al-Si alloys mainly include adhesion, brazing (or soldering) and laser sealing. Casalegno et al. [28] joined carbon fiber reinforced polymer with Al-Si alloy by three different epoxy adhesives for space applications. Gao et al. [5] studied the brazing of Si_p_/Al MMCs to Kovar alloy using active foil brazing filler metals in vacuum environment. Wang et al. [29] joined hypereutectic Al-50Si alloys by ultrasonic-assisted flux-less soldering using Zn-5Al filler metal at 420 °C in air. Xu et al. [17] realized the laser hermetic sealing of high silicon aluminum alloy CE17(Al-27Si) to CE11(Al-50Si) while the bonding of high silicon aluminum alloy CE11 to CE11 was difficult. When Si content was high, laser sealing easily leads to interface reactions and cracks. Adhesive joints have the characteristics of low strength and low working temperature, in addition, organic adhesives will age after long-term use. Ultrasonic-assisted brazing requires special equipment and the process is complicated. Therefore, brazing without ultrasound assistance was used for this research. The brazing filler materials includes brazing filler metal and brazing filler glass. The brazing filler metal needs to be used in a vacuum environment, and due to the potential difference of the metal atoms, electrochemical corrosion is likely to occur in the subsequent nickel plating and gold plating process. Therefore, this research employed low-temperature glass as brazing filler material in atmosphere.

Some ceramic materials including aluminum ceramics, sapphire, Li-Ti ferrite, and Al_2_O_3_/Cu dissimilar materials, etc. have been successfully jointed with brazing filler glass [30,31,32]. As is known, traditional low-temperature glass generally contains Pb element, which is toxic and harmful to the human body. It has been banned or restricted in electronic materials by many European and American countries. Bi and Pb are adjacent elements in the periodic table, therefore their structure and chemical properties are similar. Substituting Bi for Pb is becoming a trend. Bismuth-based glass solder has the characteristics of low softening temperature, adjustable expansion coefficient and easy formation of crystallization [33,34]. Its working temperature is generally between 420 °C and 550 °C, suitable for the welding and sealing of aluminum-based materials.

In this study, the surface of hypereutectic Al-50Si alloys was pre-oxidized to form chemical bonds similar to glass first, and then the Bi_2_O_3_-ZnO-B_2_O_3_ series of low-temperature green glass powder was used as the brazing filler glass to bond the hypereutectic Al-50Si alloys.

The purpose of this research is to provide certain theoretical support and technical reference for the joining of hypereutectic Al-Si alloys, and promote the application of which in electronic packaging, aerospace and other fields. The replacement of traditional Kovar alloy by hypereutectic Al-Si alloys is particularly urgently needed for phased array radar T/R modules. With the characteristics of environmental protection and energy saving, the brazing filler glass could realize the brazing of hypereutectic Al-50Si alloys in atmosphere environment. The brazing of hypereutectic Al-50Si alloys will lay the foundation for the further brazing of similar materials.

## 2. Materials and Methods

### 2.1. Materials Preparation and Joining Procedures

**Figure 1:**
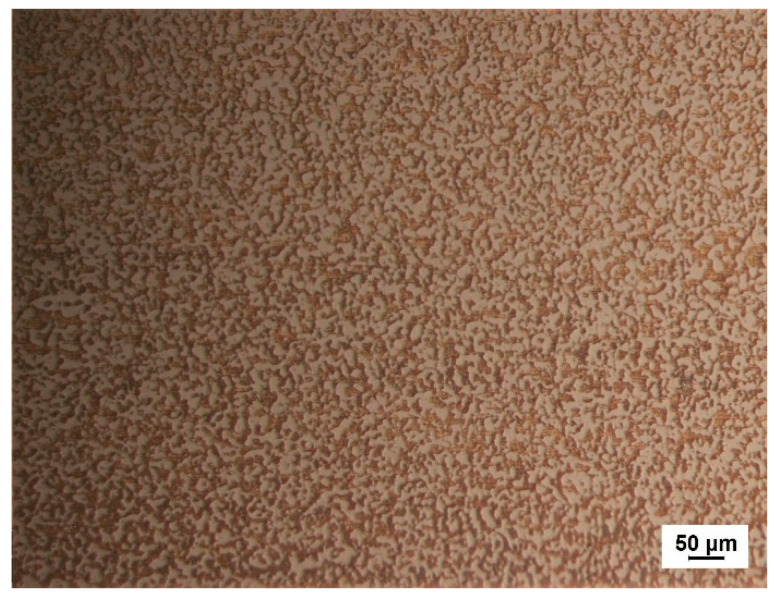
Optical microstructure of the hypereutectic Al-50Si alloys.

The commercially available hypereutectic Al-50Si alloys employed in this study was provided by the New Materials Engineering Center of Central South University, whose nominal composition was Si 50.6 wt.%, Fe 0.02 wt.%, Ca 0.01 wt.%, Sn 0.015 wt.%, Zn 0.015 wt.%, Pb 0.015 wt.%, with Al balanced. The hypereutectic Al-50Si alloys was produced using spray casting technique, whose metallographic structure is shown in Figure 1. As can be seen, silicon particles are uniformly distributed without obvious defects such as cracks, pores, voids, inclusions, etc. Tensile strength of the hypereutectic Al-50Si alloys was ~220 MPa and the coefficient of thermal expansion was ~11.5 × 10^−7^/°C. Flake samples with the size 15 × 20 × 2 mm was machined from a bulk base material. Both surfaces of the specimens were burnished by 400, 600, and 800 # metallographic abrasive papers sequentially, after that, ultrasonically cleaned in acetone for 15 min and in alcohol for 10 min to remove oil and dirt on the surface of the samples, respectively. After being dried by cold air, the processed hypereutectic Al-50Si alloys samples were put in a muffle furnace (GWL-1200) which was also used for joining tests to be pre-oxidized in the atmosphere. The surface of hypereutectic Al-50Si alloys would produce a film of Al_2_O_3_ and SiO_2_ oxides after pre-oxidation treatment, which have similar chemical bonds with glass. This would promote the wettability of the glass on the hypereutectic Al-50Si alloys and improve the joining effect. Temperature control accuracy of the furnace was ±1 °C. The samples prepared for pre-oxidation were heated up to temperature 545 °C which was maintained for 120 min at the rate of 10 °C/min and then were cooled naturally with the furnace. The samples were taken out from the furnace when the temperature was below 100 °C and placed in a closed container for later use.

**Figure 2:**
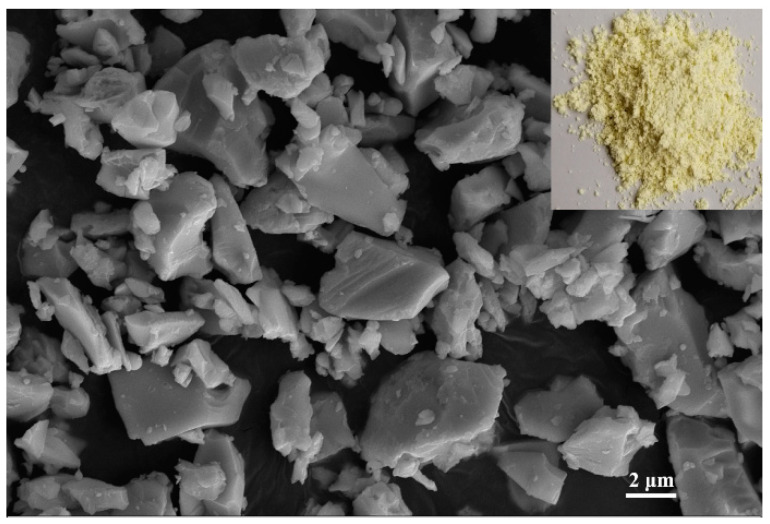
Macro morphology and microstructure of the brazing filler glass powder.

The brazing filler glass used in this investigation was the Bi_2_O_3_–B_2_O_3_–ZnO system lead-free glass produced by melt-quench method. The macro morphology and SEM image of brazing filler glass are presented in Figure 2. The chemical compositions of this brazing filler glass are shown in Table 1, which is similar to the glass used in our previous work [8]. As can be seen from Figure 2, the low-temperature glass powder is a fine yellow powder. The microscopic particle size is quite small and the maximum diameter is about 7 μm.

Brazing with paste or powdered brazing filler glass was prone to easily generate pores or adhesive residue. In this experiment, flake brazing filler glass was used for joining test. First the fixed weight glass powder was putted in a self-made mold. After compaction, it was placed in a resistance furnace (GWL-1200) for sintering. The mold was heated to 300 °C at the rate of 10 °C/min, and then at the rate of 5 °C/min to 460 °C with a heat preservation for 30 min. The flake brazing filler glass with a size of 7.5 × 7.5 × 0.5 mm was obtained. The glass column (φ5 × 3 mm) used for wetting experiment was sintered and shaped by the same sintering process as the flake brazing filler glass.

**Figure 3:**
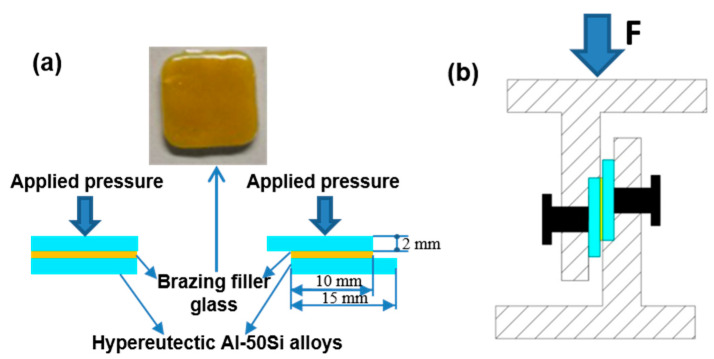
Schematic diagrams of experimental procedure: (**a**) brazing assembly method; (**b**) shear test device.

The wettability tests were implemented before joining tests. The sintered columnar glass blocks were putted at center of the hypereutectic Al-Si alloys sheets after pre-oxidation, and then the sample was heated to 475 °C, 495 °C, 515 °C, 535 °C, and 555 °C for 30 min respectively. The assembled sandwich structure specimen is shown in Figure 3a and the shear test device is shown in Figure 3b. The butt joint specimens were used for metallographic inspection and air tightness test while the lap specimens with a lap length 10 mm were adopted for shear tests. Three specimens were selected for air tightness tests and shear tests, respectively. The brazing tests were carried out in the muffle furnace which was previously used for the wetting tests in an atmospheric environment. When brazing, a pressure of 10 KPa was implemented on the combined structure.

**Figure 4:**
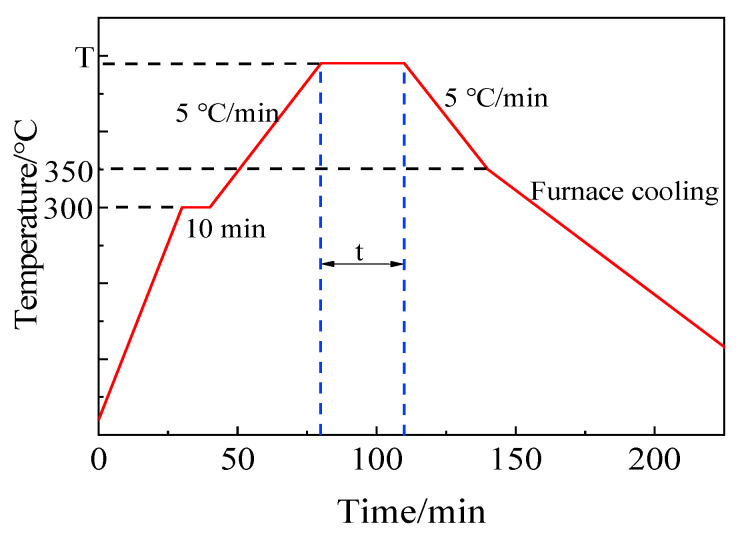
Process curve of brazing experiment.

The process curve of brazing experiment is shown in Figure 4. Firstly, to eliminate temperature gradient and maintain the consistency of temperature on specimen, the sandwich structure specimens were heated up to 300 °C at the rate of 10 °C/min, and the temperature was kept on for 10 min. Secondly, the specimens were continued to be heated up to brazing temperature for corresponding holding time. Subsequently, the specimens were slowly cooled down to 350 °C at a cooling rate 5 °C/min. At last, the specimens were cooled to room temperature in furnace naturally. The main purpose of controlling the temperature rise and fall rate in the brazing process was to reduce the brazing stress and the generation of bubbles in filler glass.

### 2.2. Characterization Methods

Thermophysical properties of the brazing filler glass were detected by differential scanning calorimetry (DSC, Q100, TA Instruments, New Castle, DE, USA). A dilatometer (DIL402C, Netzsch, Selb, Germany) was employed to evaluate the coefficients of thermal expansion (CTEs) of the brazing filler glass. Shear tests of the brazing joints were implemented by an electronic mechanical performance testing machine (CMT5105, MTS Systems (China) Co., Ltd., Shenzhen, China) at the rate of 0.2 mm/min under normal temperature. We selected three specimens under the same brazing parameter, finally average value of the three samples was adopted as shear strength. The wettability of the brazing filler glass on pre-oxidized hypereutectic Al-50Si alloys sheet was detected using an optical microscope (OLYMPUS GX51, Olympus Corporation, Tokyo, Japan). The micro morphology of the joint was observed by scanning electron microscope (SEM, Carl Zeiss NTS GmbH, Merlin Compact, Jena, Germany). Element identification and distribution were analyzed by an energy dispersive X-ray spectroscopy (EDS) attached to SEM. The phases of the glass and the joint interface were identified by X-ray diffraction (XRD, SmartLab, Rigaku Corporation, Tokyo, Japan) using Cu-Ka radiation (λ = 0.15406 nm) with the operating conditions of 40 KV and 150 mA at a scanning speed of 10°/min. Air tightness of the joint was detected by a helium leak mass spectrometer (ZQJ-530, KYKY Technology Development Ltd., Beijing, China). The worst air tightness value was adopted for three selected test specimens under the same brazing parameter.

## 3. Results and Discussion

### 3.1. Thermophysical Properties of Brazing Filler Glass

**Figure 5:**
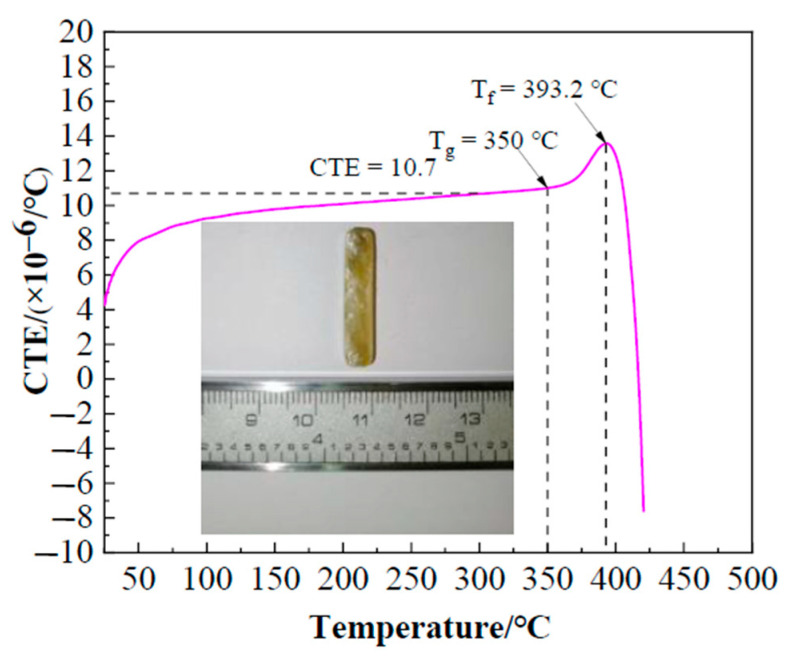
Coefficient of thermal expansion (CTE) of the brazing filler glass as function of temperature.

Figure 5 illustrates the linear thermal expansion characteristics of Bi_2_O_3_-ZnO-B_2_O_3_ system brazing filler glass as function of temperature. As can be seen, the coefficient of thermal expansion (CTE) of the glass was ~10.7 × 10^−6^/°C, which was similar to the CTE value ~11.5 × 10^−6^/°C of hypereutectic Al-50Si alloys. It is beneficial to reduce the stress caused by the thermal expansion coefficient mismatch. Also, glass transition temperature T_g_ and softening temperature T_f_ of the glass are 350 °C and 393.2 °C respectively, which can be obtained from the thermal expansion curve.

**Figure 6:**
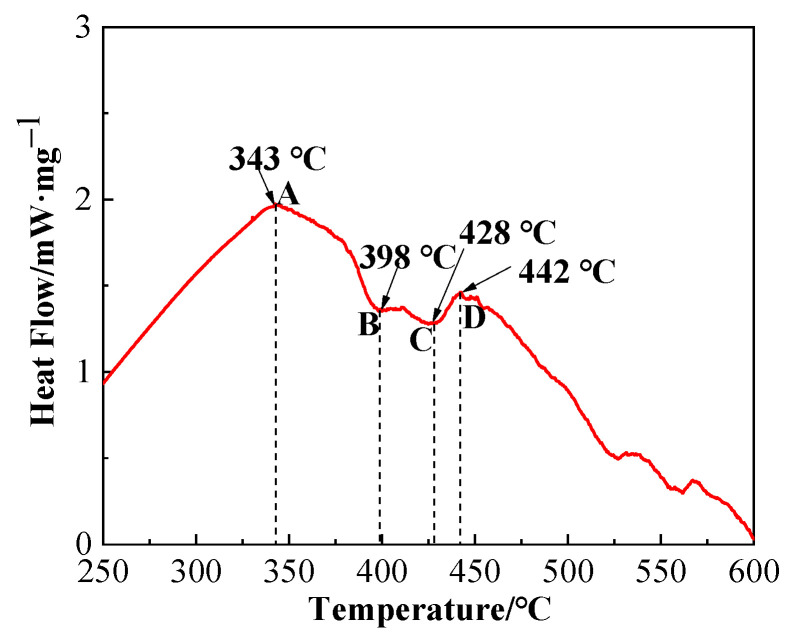
Differential scanning calorimetry (DSC) graph of the brazing filler glass.

Differential scanning calorimetry (DSC) graph of brazing filler glass is shown in Figure 6. Value and note of the representative points in DSC graph are presented in Table 2. As can be seen from Figure 6, glass exhibited a transition temperature of 343 °C and a softening temperature of 398 °C. The smile difference in characteristic temperature may be caused by device compared to the linear thermal expansion curve in Figure 5. According to comprehensive analysis, glass transition temperature T_g_ was in a range of 343–350 °C and softening temperature T_f_ was 393.2–398 °C. Therefore, the sintering temperature of flake brazing filler glass was designed from 400 to 460 °C according to comprehensive results. The tests proved that 460 °C was the best sintering temperature, and the sintered flake brazing filler glass was dense and defect-free, as shown in Figure 3a.

**Figure 7:**
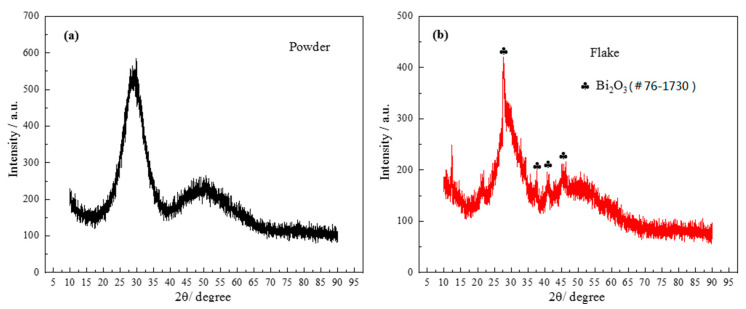
X-ray diffraction (XRD) patterns of powder and flake brazing filler glass: (**a**) powder; (**b**) flake.

As can be seen from the DSC graph, the first obvious crystallization peak appeared when the temperature was about 442 °C. In order to confirm the phase composition of the original glass powder and the sintered flake glass, the original glass powder and the sintered flake glass were analyzed by XRD respectively, as shown in Figure 7. The results showed that the original glass powder was amorphous without diffraction peaks, while some crystallization occurred in the sintered flake glass which was founded at the temperature of 460 °C for 30 min. The crystalline phase was proved to mainly be Bi_2_O_3_. Due to the small number of crystalline phases, it has little effect on the next brazing test.

**Figure 8:**
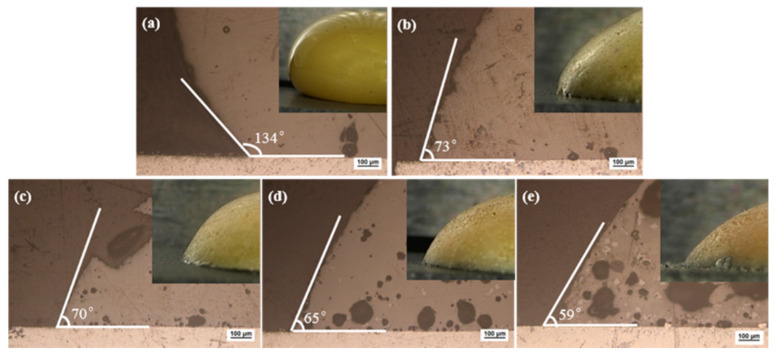
Wetting macro morphology and micro images of brazing filler glass on pre-oxidized hypereutectic Al-50Si alloys at different temperatures for 30 min: (**a**) 475 ° (**b**) 495 °C; (**c**) 515 °C; (**d**) 535 °C and (**e**) 555 °C.

To judge the brazing performance of the brazing filler metal, it is generally judged by the wettability of the brazing filler metal on the surface of the base metal, and the wetting angle can be used to characterize the wettability. It is generally believed that the brazing filler metal can wet the base metal when the wetting angle is less than 90°, and it will be better when the wetting angle is smaller. This criterion also applies to low temperature brazing filler glass [31]. Brazing temperature would be selected based on the glass wetting angle on the surface of pre-oxidized hypereutectic Al-50Si alloys. Figure 8 showed macro morphology and micro images of wetting angle for the brazing filler glass on pre-oxidized hypereutectic Al-50Si alloys from 475 °C to 555 °C with holding time 30 min. The result showed that the angle gradually decreased as brazing temperature increased. When the temperature was 475 °C, the wetting angle was 134°, indicating that the temperature was too low and not suitable for brazing. When the temperature rose to 495 °C, the wetting angle dropped rapidly from 134° to 73°. This was mainly because the wettability of the low temperature glass on the surface of pre-oxidized hypereutectic Al-50Si alloys increased rapidly as the viscosity of the low temperature glass dropped to a certain threshold. At this time, the wetting angle was lower than 90°, which made it possible to successfully braze hypereutectic Al-50Si alloys. When the temperature increased from 495 °C to 555 °C, there was a small drop of 14° for the wetting angle from 73° to 59°. Literature studies [35] have shown that it was suitable for joining of glass to metal when the wetting angle was between 45° and 90° for brazing filler glass.

As can be seen from micro images in Figure 8, crystals and pores appeared in the brazing filler glass with the increase of temperature. The number and size of crystals and pores increased as the temperature increased. When the temperature was 475 °C, there was almost no crystallization in the glass. When the temperature was 495 °C and 515 °C, a small amount of fine crystalline phases appeared in the glass. When the temperature was 535 °C and 555 °C, the size and number of crystallization phases inside the glass were significantly increased. Simultaneously, when the temperature was 475 °C and 495 °C, there were fewer pores inside the glass and the wetting interface. However, when the temperature rose to 515 °C, the pores inside the glass and the wetting interface increased significantly. As the temperature increased, the number and size of pores increased significantly.

Macro images of brazing filler glass on pre-oxidized hypereutectic Al-50Si alloys are also demonstrated in Figure 8. When the temperature was 475 °C, the glass surface was smooth, bright and shiny. As the temperature rose, the surface gradually dimmed and became rough. When the temperature was 495 °C, there was still part of the glass surface luster. However, when the temperature was 515 °C, the glass surface lost its luster and became dark completely. When the temperature exceeded 515 °C, the glass surface had obvious pinholes, and the phenomenon of “sweating” appeared. The sweating phenomenon became more obvious as the temperature rose higher, more seriously, the hypereutectic Al-50Si alloys had obvious aluminizing phenomenon when the temperature was 555 °C. The yellow color of the glass surface also gradually faded as the temperature rose. When the temperature reached 555 °C, the glass color had changed to orange.

### 3.2. Effect of Brazing Temperature on Joint Microstructure

Temperature was the most important factor affecting brazing and directly determined the performance of brazing joints. If the temperature was too low, the viscosity of the brazing filler material was too high, therefore the base material could not be wetted well. It was difficult to form an excellent brazing joint for the poor atom diffusion ability. However, when the temperature was too high, it was easy to cause excessive overflow and over burning of the brazing filler material, also defects such as pores and voids would be formed.

**Figure 9:**
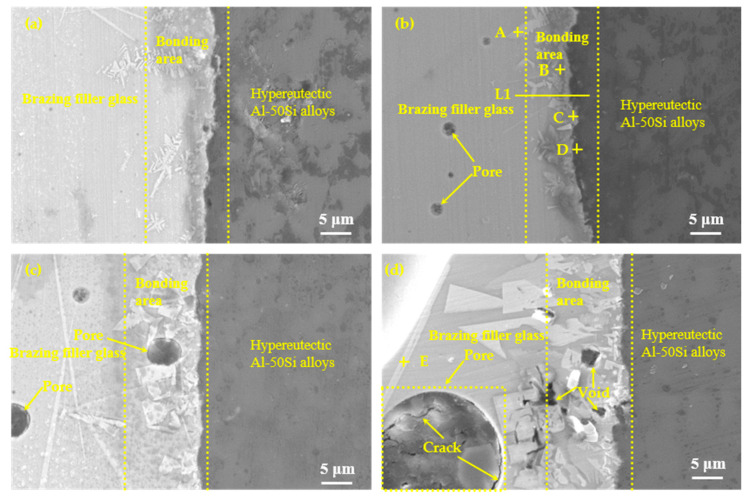
Microstructure evolution of the joints as function of temperature with holding time 30 min: (**a**) 475 °C; (**b**) 495 °C; (**c**) 515 °C and (**d**) 535 °C.

According to the results of the wettability tests shown in Figure 8, the brazing temperatures were selected as 475 °C, 495 °C, 515 °C, and 535 °C with a constant holding time of 30 min, as shown in Figure 9. It could be seen that effective brazing joints were formed under the four temperatures. The overall results of the SEM images at different temperatures showed that there was an obvious diffusion transition layer between the glass seam and the hypereutectic Al-50Si alloys when the temperature was in the range of 475–515 °C while the diffusion transition layer disappeared when the temperature was 535 °C. When the temperature was 475 °C, the width of the diffusion transition layer was about 3~10 μm with a small amount of gray snowflake-like and needle-like crystal phases generated which mainly concentrated at the joining interface or near the interface in the brazing joint. There were a small number of pores whose maximum diameter did not exceed 1 μm in the brazing joint. Meanwhile, cracks, voids and other obvious defects cannot be found in the joint. When temperature rose to 495 °C, the diffusion transition layer was narrow on average, which was about 3~5 μm. The diffusion transition layer became blurred locally and more crystalline phases formed. The snowflake-like crystalline phases gradually became irregular massive crystals. The size of the pores inside the brazing seam was obviously larger with a maximum diameter of about 2 μm. The joint was dense and there were no obvious defects such as cracks and voids. When the temperature continued to rise to 515 °C, the boundary of the diffusion transition layer became vague and the width became significantly wider, about 15 μm. There were pores with a maximum diameter of about 5 μm in the transition layer and the brazing joint. The needle-like crystal phase became significantly larger and the massive crystalline phase turned into coarse flaky phases. The number of the coarse flaky phases mainly in the transition layer increased, which would cause the performance of the brazing joint to decrease. When the temperature was 535 °C, the diffusion transition layer disappeared, and the needle-like phases and the flaky phases became larger. Meanwhile, a large pore with a diameter of about 20 μm appeared in the center of the brazing seam, in which obvious cracks originated. There were many cavities on brazing filler glass side near the edge of brazing joint, also smaller dot-like crystal phases generated on the joining interface, which would make the joint brittle and reduce the mechanical properties of the brazing seam.

It could be seen that the pores in the joint were unavoidable. For brazing filler glass, the pores continued to grow with the increase of temperature. There were two main reasons for the formation of pores. The first was that air would inevitably be entrapped in powder particles when glass powder was densified to form glass flake. On the other hand, the glass contained volatile element Zn, which was easily to volatilize from brazing filler glass forming the pores. With the increase of temperature, the partial pressure of the original pores increased and the volatilization of Zn increased, causing the pores to grow up.

**Figure 10:**
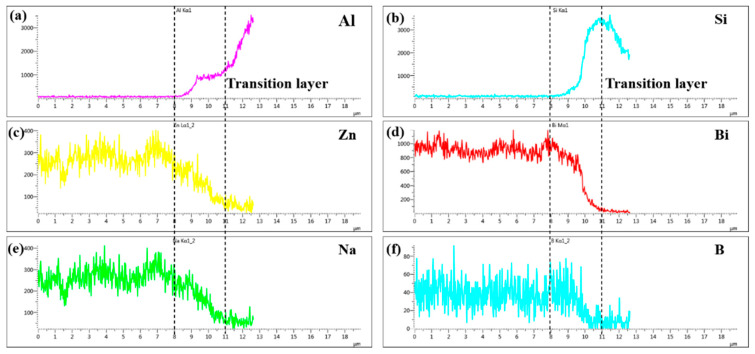
Line scanning profile of different elements at line L1 marked in Figure 9b: (**a**) Al; (**b**) 495 Si; (**c**) Zn; (**d**) Bi; (**e**) Na and (**f**) B.

To confirm the elemental composition of the typical area of the diffusion transition layer and the elemental composition of the crystalline phases, the points A, B, C, and D marked in Figure 9b and the point E marked in Figure 9d were scanned by an energy dispersive X-ray spectroscopy (EDS). To understand the diffusion of elements in the transition diffusion layer, the energy spectrum analysis line scan was performed on the position L1 marked in Figure 9b. The point scan results are shown in Table 3 and the line scan results are shown in Figure 10, respectively. The main components of the bulk crystal phase point A were elements Bi, B and O, and which of the needle crystal phase point E were elements Bi, B, O, and Zn. The points B, C, and D were in the transition regions between the brazing filler glass and hypereutectic Al-50Si alloys. It could be seen that the Bi element content at point B was up to 61.0 wt.% while the content of which at point C was reduced to 46.8 wt.%. However, the Bi element content at point D dropped sharply to only 1.4 wt.%. Similarly, the content of elements Zn and Na also showed a downward trend in the diffusion transition zone from left to right. On the contrary, the content of elements Al, Si, and B gradually increased, indicating that the elements in the interface transition zone had undergone significant mutual diffusion. It was worth noting that the content of element B should be reduced theoretically from left to right as B_2_O_3_ was one of the main components of the brazing filler glass, however, the content of element B increased gradually. The reason may be that the size of atom B was very small, resulting in a more obvious diffusion and aggregation effect at the interface.

Figure 10 was the line scanning profile of different elements at line L1 marked in Figure 9b, which was made at 495 °C with a holding time 30 min. As can be seen, the diffusion transition layer with thickness of 3 μm was marked with dotted line in Figure 10. The elements Al, Si, Zn, Bi, Na and B had an obvious concentration gradient distribution. The concentration distribution of elements Zn, Bi, Na gradually decreased while which of elements Al and Si gradually increased. However, the concentration distribution of element B first increased and then decreased. The results were consistent with the point scan results in Table 3. It was worth noting that the diffusion distance of Zn, Na, and B exceeded the diffusion transition layer while the diffusion of Al, Si, and Bi was limited in the diffusion transition layer, indicating that elements Zn, Na, and B had diffused over a long distance.

**Figure 11:**
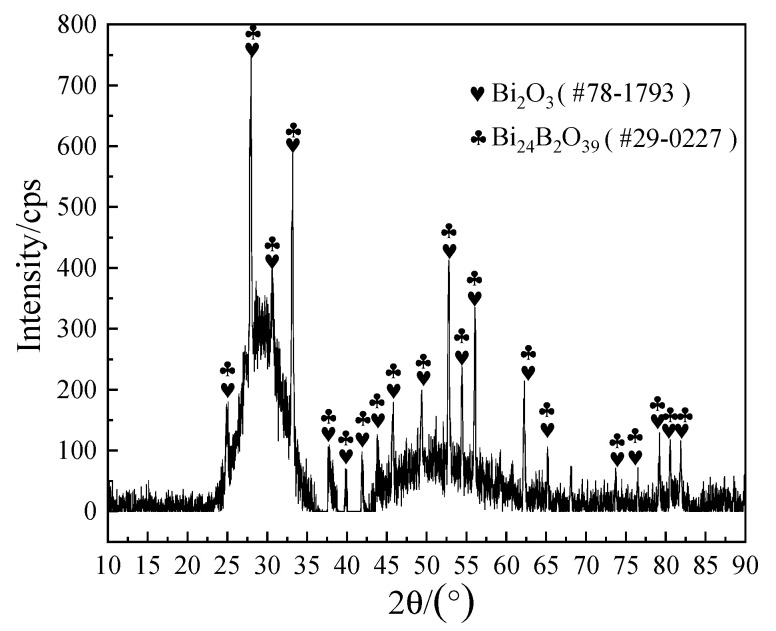
XRD patterns of the brazing joint fracture from Figure 9b.

To further confirm the composition of the crystalline phases, XRD analysis was implemented and the analysis results are shown in Figure 11. Combined with the EDS results in Table 3, it could be determined that the bulk crystal phase A was Bi_24_B_2_O_39_, which was consistent with the report in the literature [34], while the acicular phase E may not be detected by XRD because of its low content.

**Figure 12:**
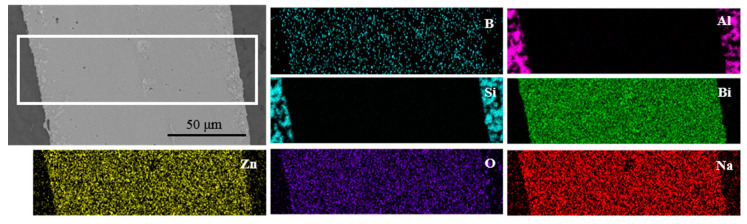
EDS maps of different elements in joint made at brazing temperature of 495 °C for 30 min.

To investigate the element distribution, the energy dispersive X-ray maps were captured on the joint which was made at brazing temperature of 495 °C for 30 min, as shown in Figure 12. It could be seen that the elements Bi, Si, and Al did not diffuse obviously on the surface scan map with a short diffusion distance. However, the elements Na, Zn, O, and B had obvious diffusion phenomena on both sides of the brazing joint, which showed that the Na, Zn, O, and B elements had a long-distance diffusion. It was consistent with the results as displayed in line scanning profile in Figure 10.

### 3.3. Effect of Brazing Time on Joint Microstructure

**Figure 13:**
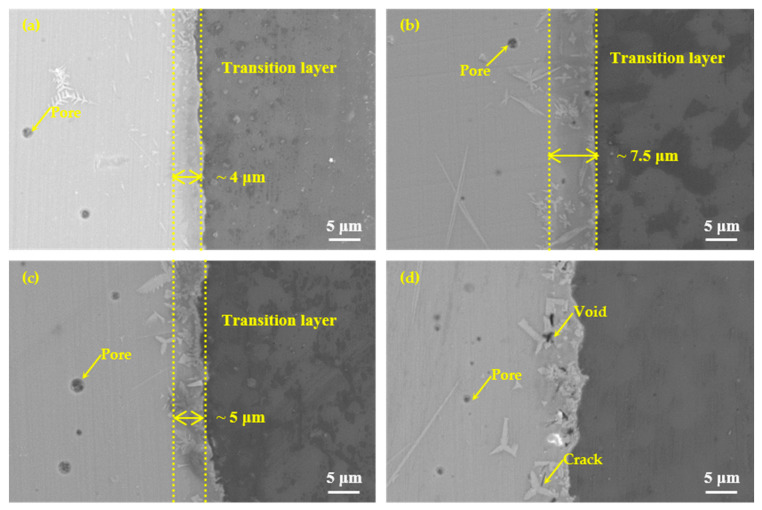
Microstructure evolution of the joints at the brazing temperature 495 °C for different brazing times: (**a**) 10 min; (**b**) 20 min; (**c**) 30 min and (**d**) 40 min.

In addition to brazing temperature, brazing time was also one of the important factors affecting the performance of brazing joints. To better understand the joining mechanism of the brazing filler glass to the hypereutectic Al-50Si alloys, brazing time effect on the joint microstructure evolution was also studied, as shown in Figure 13. It showed microstructure evolution when brazing temperature was 495 °C with different holding times 10 min, 20 min, 30 min and 40 min, respectively. When the holding time was 10 min, there were a few pores with a maximum diameter of about 2 μm and a small snowflake crystal phase in the center of the brazing joint, also a small amount of fine crystalline phases generated in the diffusion transition layer. However, no pores were formed at the interface, mainly because the holding time was short. The glass viscosity was relatively high, therefore, the pores at the interface were difficult to grow. A blurred diffusion transition layer of about 4 μm existed at the interface. When the holding time was 20 min, the diffusion transition layer obviously increased to 7.5 μm. There were pores with also a maximum diameter of about 2 μm in the center of the brazing joint and diffusion transition layer. The number of pores increased and the diffusion transition layer was still blurred. The number and size of crystalline phases increased significantly. When the holding time was 30 min, the width of the diffusion transition layer was about 5 μm, which was obviously black, dense and clear. The pores with a maximum diameter of about 3 μm only appeared in the center of the brazing seam. The crystalline phases whose size had an increase were mainly in the diffusion transition layer. When the holding time was 40 min, the diffusion transition layer almost disappeared. The sizes of the flaky and needle-like crystalline phases had become significantly larger, and the numbers of which also increased. Interestingly, the number of pores had increased. However, the maximum diameter was decreased to about 1 μm. This was mainly because large pores could volatilize and disappear as the holding time increased. Obvious voids and cracks appeared in the diffusion transition layer, which would reduce the air tightness and strength of the joint.

Generally speaking, as the holding time increased, the size and number of pores did not change significantly. The width of the diffusion transition layer at the interface changed significantly. It first became bigger, then narrower, and finally disappeared, indicating that the diffusion degree of elements had a greater relationship with the holding time.

### 3.4. Shear Strength Test of Brazing Joints

**Figure 14:**
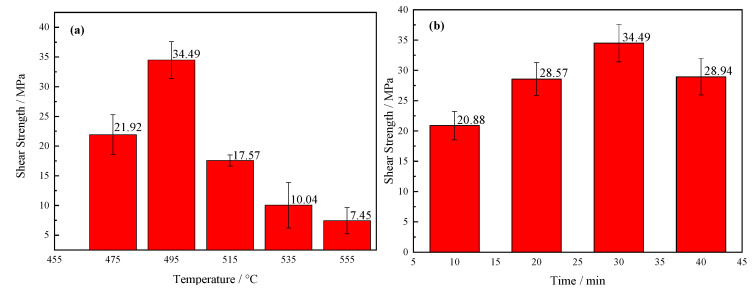
Shear strength of the brazing joints as a function of: (**a**) brazing temperature; (**b**) brazing time.

Mechanical properties were the most intuitive means for characterization of the quality for a brazing joint. To find the best process of brazing, this research separately studied the influence of brazing temperature and holding time on the shear strength of brazing joints. First, the holding time 30 min was selected, and the brazing temperature was 475 °C, 495 °C, 515 °C, 535 °C, and 535 °C, respectively, as shown in Figure 14a. The shear strength of the joint first increased at first, and then it decreased as increase of brazing temperature. When temperature was 495 °C, the average strength reached the highest value of 34.49 MPa. As can be seen from the SEM images in Figure 7, when the temperature was low, the viscosity of the brazing filler glass was relatively high, and the diffusion ability of the element atoms was weak. Although an effective joint was formed, the bonding was not excellent sufficiently due to insufficient diffusion of the elements. Therefore, the shear strength was low. As the temperature rose, the size of the crystalline phases gradually became larger and the quantity of which increased. When the temperature was 495 °C, the elements diffusion was relatively sufficient, and the smaller crystalline phases had the effect of strengthening phases. As a result, the joint was dense and the pores less, therefore the joint strength was high. When the temperature reached 515 °C, the density of the brazing filler glass decreased due to the increased number and large size of the pores. Meanwhile, the large pores at the interface reduced joint strength. When the temperature was over 535 °C, the size of the pores and crystalline phase accelerated to grow up. In addition, more cracks and cavities were formed in the diffusion transition layer, and the brazing filler glass became loose and brittle. There were also obvious cracks inside the large pores, resulting in a sharp drop for the strength of the brazing joint.

Correspondingly, the effect of holding time on the strength of joints was also investigated, as shown in Figure 14b. The brazing temperature was 495 °C and the holding time was 10 min, 20 min, 30 min and 40 min, respectively. On the whole, the joint strength changed within a small range. The value of shear strength was the highest when the holding time was 30 min while it was the lowest when the holding time 10 min. When the holding time was short, the brazing filler glass had a high viscosity and the diffusion degree of elements was low. As a consequence, the joint with poor shear strength was obtained. When the holding time was 40 min, there were certain cavities and small cracks in the diffusion transition layer of the joint, however, the strength did not drop sharply. The main reason was that the elements diffusion at the bonding interface was more sufficient, and the transition layer was no longer visible, which would increase shear strength of the joint to a degree.

**Figure 15:**
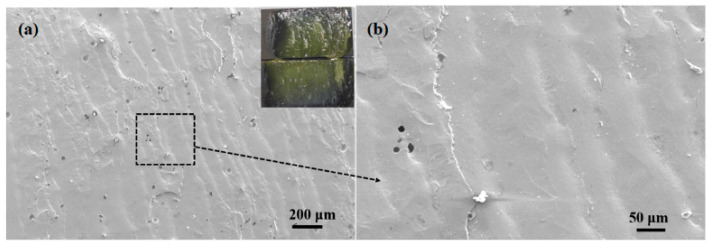
Fracture morphology of the joint made at brazing temperature of 495 °C for 30 min: (**a**) macro and low magnification morphology; (**b**) magnification morphology 3.5. Air Tightness Test of Brazing Joints.

To investigate the fracture mechanism of brazing joint, macroscopic and microscopic observation and analysis of the joint fracture made at 495 °C for 30 min were carried out, as shown in Figure 15. It could be seen that the joint fractured from the inside of the brazing filler glass during shearing, and there were some small-sized pores inside the glass which had little effect on the performance of the joint. No crystalline phase was found, indicating that the number of crystalline phases in the brazing filler glass was relatively small under this process. The overall wave-shaped deformation of the brazing filler glass on the fracture indicated that the filler glass had better viscoelasticity and less brittleness at this temperature. Therefore, the shear strength of the joint was higher under this process.

For electronic packaging devices, air tightness was one of the most important parameters. The main purpose of ensuring air tightness was to prevent the internal chips from being corroded by atmospheric oxygen, water vapor and other media, maintaining the efficient work of electronic packaging products. This research was mainly aimed to the application of novel brazing filler glass on joining of hypereutectic Al-50Si alloys, which was an attractive candidate in electronic packaging materials. The research of joint airtightness was of great value to popularize the application of hypereutectic Al-50Si alloys in electronic packaging and aerospace fields. Based on Chinese national standard GB 5594. 1–85, the air tightness leak rate should not be higher than 10 × 10^−8^ Pa m^3^/s. Table 4 displays airtightness of the joint at different brazing temperatures for 30 min, and each value was the worst one in three samples under the same brazing process. Table 5 displays air tightness of the joints at the temperature of 495 °C for different holding times. Similarly, each value was also the worst one in three selected samples.

As shown in Table 4, when the temperature was 495 °C, the air tightness leakage rate was 1.0 × 10^−10^ Pa m^3^/s, which was the best in this study. When the temperature exceeded 535 °C, the air tightness was 5.6 × 10^−7^ Pa ^3^/s, which was below the national standard, therefore, the air tightness was unqualified. As can be seen from Figure 9d, there was a large pore with a diameter of about 20 μm in the center of the joint under this process, and many cracks and voids generated near the joint interface, which were the main reason for the unqualified air tightness. As can be seen from Table 5, it could be found that the air tightness leakage rate was the lowest when time was 30 min. Meanwhile, the air tightness leakage rate was 3.4 × 10^−9^ Pa m^3^/s when time was 40 min, which was the worst. However, it also could meet the national standard. The results indicated that air tightness was not sensitive to brazing time. The air tightness results were basically consistent with the shear strength results in this study. That is to say, there was a good correspondence between the quality of air tightness and the strength of the joints, which showed that studying the shear strength of the joints had a good guiding effect on the quality of the joint air tightness.

## 4. Conclusions

With the characteristics of low temperature and lead-free, the brazing filler glass successfully joined hypereutectic Al-50Si alloys together in atmosphere. Thermophysical analysis, microstructure evolution observation, shearing strength tests, and leakage rate tests of air tightness were implemented to characterize the performance of brazing filler glass and the brazing joints. The main observations are summarized as follows:

(1) The coefficient of thermal expansion of the brazing filler glass was ~10.7 × 10^−6^/°C, which was similar to the value 11.5 × 10^−6^/°C of used hypereutectic Al-50Si alloys. The matching of thermal expansion coefficient between brazing filler glass and hypereutectic Al-50Si alloys was beneficial for reducing joint stress and improving the joining quality. The glass transition temperature T_g_ was in a range of 343–350 °C and softening temperature T_f_ was in a range of 393.2–398 °C. Keeping a holding time of 30 min as a constant, the wetting angle of brazing filler glass on pre-oxidized hypereutectic Al-50Si alloys gradually decreased from 73° to 59° when the temperature changed from 495 °C to 555 °C.
(2) Using Bi_2_O_3_-ZnO-B_2_O_3_ system brazing filler glass could achieve the joining of hypereutectic Al-50Si alloys. Brazing temperature and time had significant effects on joint microstructure. The rational brazing joint was obtained at brazing temperature of 495 °C for 30 min. The joint could reach the maximum shear strength of 34.49 MPa and a lower air tightness leakage rate of 1.0 × 10^−10^ Pa m^3^/s. The air tightness can meet the requirements of modern electronic packaging, which is essential for electronic devices, especially in T/R modules of phased array radar.
(3) During brazing process, crystallization phenomenon of the brazing filler glass appeared. The crystal products including Bi_24_B_2_O_39_ and Bi_2_O_3_ which played a role of reinforcement in joint. Increasing the brazing temperature or brazing time, the size and number of crystalline phases increased. Meanwhile, the size and number of pores in joint increased obviously with temperature rising while they were less time sensitive.
(4) The joint made at brazing temperature of 495 °C for 30 min fractured from the inside of brazing filler glass. There were some small-sized pores in glass and no crystalline phase was found. The brazing temperature had a greater effect on joint air tightness compared with the brazing time. The formation of diffusion transition layer with thickness of 3 μm was the key to form an effective joint. When the brazing temperature exceeded 535 °C or the brazing time reached 40 min, the diffusion transition layer disappeared. The local aggregation of the elements Al, Si, Zn, Bi, O, Na and B at the interface between the glass brazing seam and the base metal was the direct cause of the formation of the transition diffusion layer. When the temperature rose, the elements diffused more fully, and therefore the transition diffusion layer disappeared.

With the successful joining of hypereutectic Al-50Si alloys using lead-free brazing filler glass in air, the joining between Al-50Si alloys and electronical glass is promising in the near future. To optimize joining process and deeply understand the joining mechanism between metal and glass, other systems for joining low-temperature glass to hypereutectic Al-50Si alloys are necessary in the future.

## Figures and Tables

**Table 1:** Chemical compositions of the brazing filler glass powder (in wt.%).

Composition	Bi_2_O_3_	ZnO	B_2_O_3_	Na_2_CO_3_	SiO_2_
Content	70–80	0–10	0–5	0–5	0–5

**Table 2:** Description of feature points in Figure 6.

Point	Temperature/°C	Description
A	343	Glass transition point
B	398	Glass softening point
C	428	Crystallization start temperature
D	442	Crystallization peak temperature

**Table 3:** Point scan analysis of the points marked in Figure 9b,d (in wt.%).

Point	Al	Si	Na	O	Bi	B	Zn
A	-	0.7	-	9.4	84.2	5.0	0.7
B	-	0.7	2.3	15.7	61.0	9.0	11.3
C	2.5	15.1	2.1	13.8	46.8	11.9	7.8
D	15.1	45.1	0.4	14.2	1.4	23.1	0.7
E	-	0.3	1.0	13.7	72.7	8.1	4.2

**Table 4:** Air tightness of the joints at different temperatures for 30 min.

Temperatures/°C	475	495	515	535
Leakage rate/Pa m^3^/s	8.0 × 10^−9^	1.0 × 10^−10^	6.2 × 10^−^^8^	5.6 × 10^−7^

**Table 5:** Air tightness of the joints at brazing temperature of 495 °C for different holding times.

Times/min	10	20	30	40
Leakage rate/Pa m^3^/s	1.2 × 10^−9^	3.8 × 10^−10^	1.0 × 10^−10^	3.4 × 10^−9^
